# Hyodeoxycholic Acid Suppresses High-Fat-Diet–Promoted MC38-Syngeneic Colorectal Tumor Growth via Bile Acid Remodeling and Microbiota Modulation

**DOI:** 10.3390/nu17243939

**Published:** 2025-12-17

**Authors:** Jialing He, Meng Duan, Yuwen Shi, Simayi Halizere, Ningxin Chen, Yating Yang, Congcong Wang, Jinhua Lin, Wei He, Shankuan Zhu, Fei Yang

**Affiliations:** 1 Department of Nutrition and Food Hygiene, Children’s Hospital, Zhejiang University School of Medicine, National Clinical Research Center for Children and Adolescents’ Health and Disease, Hangzhou 310058, China; hejialing@zju.edu.cn (J.H.); duanmeng@zju.edu.cn (M.D.); 22418009@zju.edu.cn (Y.S.); 3180102244@zju.edu.cn (S.H.); 22318289@zju.edu.cn (N.C.); yangyating@zju.edu.cn (Y.Y.); congcong.wang@helmholtz-munich.de (C.W.); linjinhua@xmmc.edu.cn (J.L.); zjuhewei@zju.edu.cn (W.H.); 2 Chronic Disease Research Institute, School of Public Health, Zhejiang University School of Medicine, Hangzhou 310058, China; 3 Binjiang Institute of Zhejiang University, Hangzhou 310053, China

**Keywords:** colorectal tumors, hyodeoxycholic acid, obesity, gut microbiota

## Abstract

Background: Studies have shown that obesity contributes to colorectal tumors (CRC). Hyodeoxycholic acid (HDCA) has been found to have a therapeutic effect on obesity-related diseases such as nonalcoholic fatty liver (NAFLD). However, there are still no studies revealing whether HDCA has effects on CRC, which may suggest new uses for HDCA. Methods: C57BL/6 mice fed with high-fat diet supplemented with 0.5% HDCA were injected with MC38 cells subcutaneously to construct the subcutaneous metastasis model of CRC. The trend of body weight and tumor volume were evaluated, and blood metabolites and gut microbiota sequencing were analyzed. Results: Compared with HFD-fed mice, HDCA-treated mice had higher fecal and serum HDCA levels. After tumor inoculation, the HDCA mice had smaller subcutaneous tumor volumes, as well as higher HDCA and THDCA levels in feces and blood. Blood metabolomics revealed significant enrichment in pathways of bile secretion, arachidonic acid metabolism, primary bile acid metabolism, and taurine and hypotaurine metabolism. Analysis of gut microbiota at the completion of obesity modeling revealed the Chao1 index of the feces being lower in the HDCA mice. The relative abundance of a total of nine genera were significantly higher and eighteen genera were lower. The KEGG results indicated significant upregulation of nine metabolic pathways and downregulation of sixteen metabolic pathways. Conclusions: HDCA intake ameliorates HFD-induced obesity phenotype, inhibiting colorectal tumor growth in mice, and decreases the abundance of gut microbiota. Gut microbiota affected by HDCA may participate in metabolism-related effects through circulation, which might be one way that HDCA affects colorectal tumors.

## 1. Introduction

A high-fat diet (HFD) is characterized by excessive fat intake and insufficient levels of vitamins, fiber, and other essential nutrients. HFD not only leads to obesity [1] but is also associated with chronic diseases such as diabetes, hypertension, neurodegenerative diseases, as well as some cancers such as prostate cancer and digestive tract tumors [2,3,4].

CRC is one of the most common gastrointestinal malignancies and the second leading cause of cancer-related deaths worldwide, affecting approximately 2 million people globally [5]. The number of new cases of colorectal cancer is gradually increasing and is expected to reach 3.2 million by 2040 [6]. The incidence of colorectal cancer varies significantly, with a higher rate in high-income countries, including most European countries, North America, etc. [7]. The dietary pattern in these countries mainly consists of low dietary fiber and high levels of meat intake. This may explain part of the causes related to the current incidence of CRC. Obesity is an independent risk factor for CRC, and the epidemiologic study reported significant associations between BMI and CRC risk [8,9]. The potential mechanism of a high-fat diet promoting the occurrence and development of colorectal cancer is complex. Dietary fat intake affects bile acid synthesis and physiological process of bile discharge. Research has found that long-term intake of a high-fat diet will increase the synthesis of bile acids and the content of hydrophobic bile acids, which will increase intestinal permeability. However, hydrophilic bile acids such as ursodeoxycholic acid have the opposite effect [10].

Our previous research found that the content of ten secondary bile acids decreased in the feces of HFD-induced mice. Further intervention experiments confirmed that hyodeoxycholic acid (HDCA) can inhibit obesity induced by a high-fat diet [11]. HDCA is a kind of natural secondary bile acid which has been proven to play an important role in improving atherosclerosis [12], reducing nonalcoholic fatty liver disease (NAFLD) [13], and has important prospects in the treatment of human fatty liver. Study showed that HDCA treatment inhibited the proliferative capacity of CRC cells through the FXR/EREG/EGFR axis [14], but the role of HDCA in promoting the progression of CRC through a high-fat diet is not fully clear. This study aims to elucidate, from a multi-omics perspective, the mechanisms by which HDCA modulates the progression of colorectal cancer under a high-fat diet. Our findings are expected to provide potential therapeutic targets for obese patients with CRC, advance the understanding of obesity-related tumorigenesis, and enrich current knowledge on the role of intestinal metabolites in cancer prevention and treatment.

## 2. Results

### 2.1. Supplementation with Hyodeoxycholic Acid Inhibited Colon Cancer Progression Promoted by a High-Fat Diet

**Figure 1:**
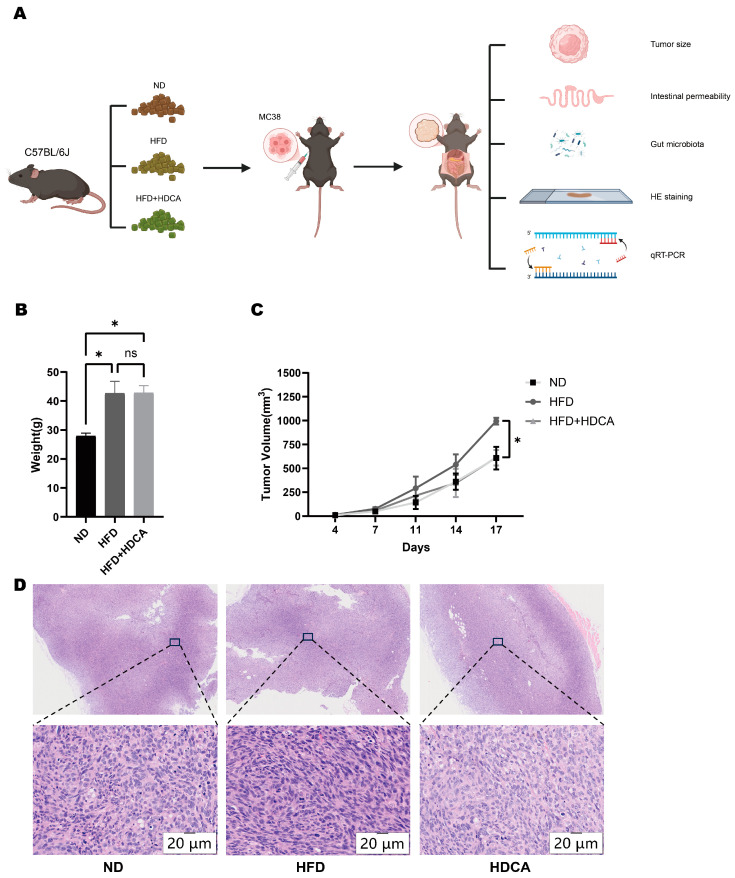
Body weight and tumor size changes in mice co-fed by HFD and HDCA. (**A**) Fed C57BL/6 mice a normal diet (ND), a high-fat diet (HFD), or a combination of high-fat diet (HFD) and hyodeoxycholic acid (HDCA). Inoculate MC38 colon cancer cells into the axillary to establish a subcutaneous tumor model of MC38 colon cancer cells. Measure tumor size, test intestinal permeability, test gut microbiota, stain tumor sections, and perform qRT-PCR. Created with Biorender.com. (**B**) Mice body weight before MC38 cell inoculation (*, *p* < 0.05; ns, no significant difference). (**C**) Tumor size by tumor inoculation days (*, *p* < 0.05, ND vs. HFD, HFD+HDCA vs. HFD). (**D**) HE staining of mouse tumors (20 μm, scanned under 200 magnification).

To determine the role of HDCA in HFD-induced tumors, we fed mice with HDCA+HFD/HFD to establish tumor models. Since HDCA can inhibit the weight gain induced by HFD in mice, in order to rule out the possibility that HDCA exerts its effect on tumors by reducing the body weight of mice, mice with similar body weights in the HFD+HDCA group and the HFD group were selected for axillary inoculation of MC38 colon cancer cells to construct a transplant tumor model (Figure 1A). It was found that mice consuming a high-fat diet and a high-fat diet containing HDCA had significantly higher body weights than mice consuming a normal diet (Figure 1B). Supplementation with HDCA inhibited HFD-induced, obesity-promoted colon cancer tumor growth (Figure 1C). After HE staining of the tumor of the mice, it was observed that compared with the HFD+HDCA group, the nuclear size of the tumor cells in the HFD group mice varied more greatly, indicating that the tumor cells had higher heterogeneity and instability (Figure 1D).

### 2.2. HDCA Modulates Bile Acid Metabolism Disorders Induced by High-Fat Diet in Mice

**Figure 2:**
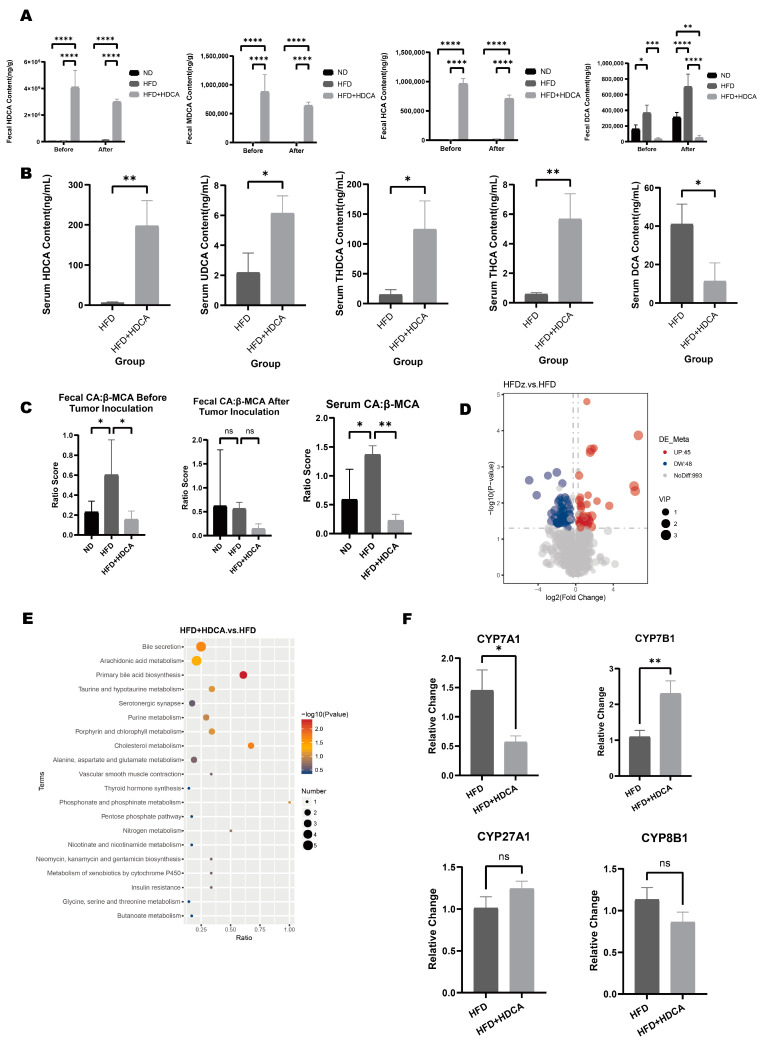
Bile acid metabolism results. (**A**) Differential bile acid content in mice feces of HDCA, MDCA, HCA, and DCA before and after MC38 cell inoculation (*, *p* < 0.05; **, *p* < 0.01; ***, *p* < 0.001; ****, *p* < 0.0001). (**B**) Different bile acid content in mice serum of HDCA, UDCA, THDCA, THCA, and DCA after MC38 cell inoculation (*, *p* < 0.05; **, *p* < 0.01). (**C**) Ratio of CA to β-MCA in mice feces before and after MC38 cell inoculation; ratio of CA to β-MCA in mouse serum after MC38 cell inoculation (*, *p* < 0.05; **, *p* < 0.01; ns, *p* > 0.05). (**D**) Volcano plot of differential metabolites (HFD+HDCA vs. HFD). (**E**) KEGG enrichment scatterplot (HFD+HDCA vs. HFD). (**F**) qRT-PCR for bile acid synthase CYP7A1, CYP7B1, CYP27A1, CYP8B1 in mice livers (*n* = 8 in each group; *, *p* < 0.05; **, *p* < 0.01; ns, *p* > 0.05).

Fecal bile acid concentrations in the ND group, the HFD group and the HFD+HDCA group were examined by targeted bile acid metabolomics, and fecal and serum bile acid concentrations were examined after MC38 cell inoculation. The results showed that the concentration of HDCA, HCA, and MDCA was increased, and the concentration of DCA was decreased before and after MC38 cell inoculation in the feces of mice in the HFD+HDCA group, compared with that in the HFD group (Figure 2A). After colon cancer cell inoculation, the serum concentrations of HDCA, UDCA, THDCA, and THCA were increased, and the concentration of DCA was lowered in the group of mice that were co-fed a high-fat diet with HDCA (Figure 2B). To test whether bile acid synthesis shifted from the classical pathway to the alternative pathway, the fecal and serum CA to β-MCA ratios were calculated. The results showed that the ratio of CA to β-MCA was significantly lower in mice in the HFD+HDCA group compared to the HFD group both before and after MC38 colon cancer cell inoculation (Figure 2C).

Our research also conducted untargeted metabolomics of blood. There are 45 metabolites that significantly increased while 48 metabolites significantly decreased in the HFD+HDCA group compared with the HFD group (Figure 2D). The KEGG enrichment analysis showed significant enrichment in pathways of bile secretion, arachidonic acid metabolism, primary bile acid metabolism, and taurine and hypotaurine metabolism. (Figure 2E).

To further explore the changes in bile acid metabolic pathways in mice, qRT-PCR was used to detect the expressions of key enzymes CYP7A1, CYP7B1, CYP8B1, and CYP27A1 for bile acid synthesis in the liver of mice. The results showed that the relative expression level of CYP7B1 in the liver of mice in the HFD+HDCA group was significantly higher, and CYP7A1 was significantly lower compared with the HFD group (Figure 2F). The results above suggested that HDCA could modulate high-fat diet-induced disorders of bile acid metabolism and related pathway changes. Intake of HDCA had a tendency to promote the transformation of bile acid metabolism from the classical pathway to the alternative pathway.

### 2.3. Hyodeoxycholic Acid Inhibits High-Fat-Diet-Promoted Colon Cancer Progression in Mice via Deoxycholic Acid

**Figure 3:**
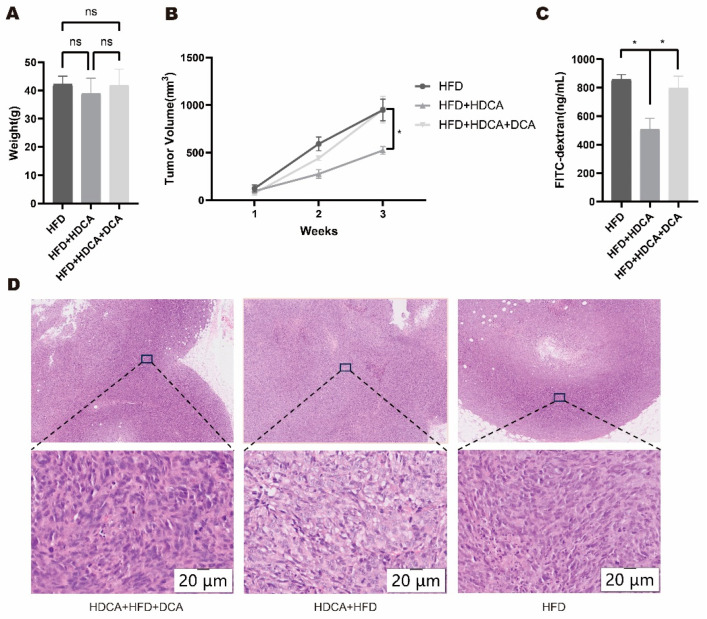
Body weight and tumor size changes in mice co-fed by HFD, HDCA, and DCA. (**A**) Co-feeding C57BL/6 mice with deoxycholic acid (DCA), hyodeoxycholic acid (HDCA), and high-fat diet (HFD), mice body weight before establishing the MC38 colon cancer subcutaneous tumor model (ns, no significant difference). High-fat diet group (HFD); hyodeoxycholic acid and high-fat diet co-feeding group (HFD+HDCA); hyodeoxycholic acid, high-fat diet, and deoxycholic acid co-feeding group (HFD+HDCA+DCA). (**B**) Established an MC38 colon cancer subcutaneous tumor model and compared tumor size (*, *p* < 0.05). (**C**) Intestinal permeability experiments were conducted to assess colonic barrier permeability in mice (*, *p* < 0.05, HFD vs. HFD+HDCA, HFD+HDCA vs. HFD+HDCA+DCA). (**D**) HE staining of mouse tumors (20 μm, scanned under 200 magnification).

To verify whether HDCA regulates colon cancer progression via DCA in high-fat dietary states, mice were co-fed with HDCA and HFD along with drinking water containing 0.2% DCA. Mice with similar body weights in the HFD group, the HFD+HDCA group, and the HFD+HDCA+DCA group were selected for axillary inoculation of MC38 colon cancer cells to construct a transplant tumor model. The results showed that there was no significant difference in body weights of mice in the HFD group, the HFD+HDCA group, and the HFD+HDCA+DCA group (Figure 3A). Mice in the HFD+HDCA group had smaller tumor volumes compared with the HFD group. Supplementation of DCA drinking water counteracted the colon cancer growth inhibited by HDCA (Figure 3B) and reversed the improvement of intestinal permeability induced by HDCA (Figure 3C). After HE staining of the tumor of the mice, it was observed that compared with the HFD+HDCA group, the nuclear size of the tumor cells in the HFD+HDCA+DCA group mice varied more greatly, indicating that the tumor cells had higher heterogeneity and instability (Figure 3D).

### 2.4. Hyodeoxycholic Acid Regulated the Composition of Gut Microbiota

**Figure 4:**
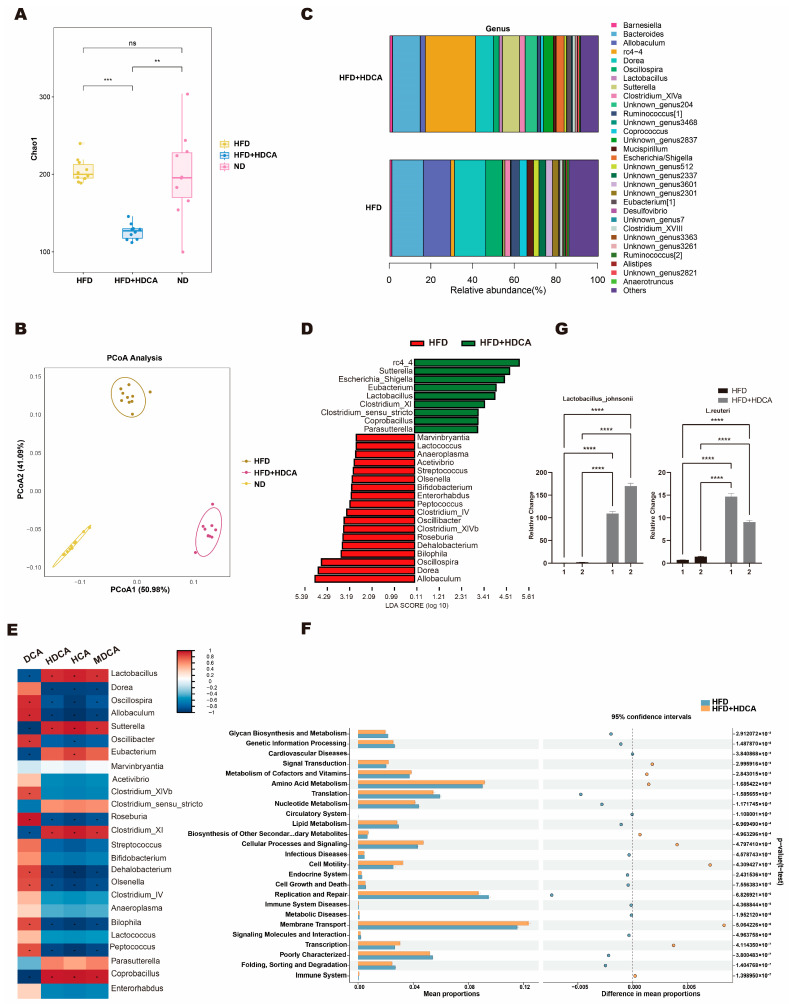
Gut microbiota results. (**A**) Chao1 value of mouse microbiota, (**, *p* < 0.01; ***, *p* < 0.001; ns, *p* > 0.05). (**B**) Beta diversity analysis based on Bray–Curtis (permutation multivariate analysis of variance, PERMANOVA, *p* < 0.01). (**C**) Gut species composition at the genus level in HFD and HFD+HDCA mice. (**D**) LEfSe differential analysis at genus level. (**E**) Analysis of the correlation between differential gut microbiota and differential bile acids before MC38 cell inoculation. (**F**) PICRUSt2 Function prediction of gut microbiota (KEGG level 2) (HFD+HDCA vs. HFD). (**G**) qRT-PCR results for *Lactobacillus johnsonii* and *L. reuteri* in the feces of mice in the HFD group and the HFD+HDCA group (****, *p* < 0.0001).

We further investigated the effect of hyodeoxycholic acid supplementation on the gut microbiota composition of obese mice by 5R 16S rRNA gene sequencing. The results of the Chao1 index in alpha diversity showed that there was no significant difference in species richness in the fecal microbiota of mice in the ND and the HFD groups. The abundance was decreased in mice co-fed with hyodeoxycholic acid and a high-fat diet (Figure 4A). Beta diversity analysis showed a significant difference between the HFD and the ND groups. There was a significant alteration in the structure of the intestinal microorganisms of mice co-fed with hyodeoxycholic acid and a high-fat diet (Figure 4B).

Analyzing the composition of mice gut microbiota structure, at the genus level, in mice in the ND group showed a significant increase in *Barnesiella* compared with the HFD group and the HFD+HDCA group. Compared with the HFD group, the relative abundance of *Lactobacillus*, *Sutterella*, etc. in the HFD+HDCA group of mice was significantly increased, and the relative abundance of *Allobaculum*, *Mucispirillum, etc. was* significantly decreased (Figure 4C).

LEfSe (LDA Effect Size) difference analysis revealed that the dominant genera in the HFD+HDCA group included *Lactobacillus*, *Sutterella*, *Eubacterium*, etc.; the dominant genera in the HFD group included *Allobaculum*, *Dorea*, *Bilophila*, etc. (Figure 4D).

Correlation analysis revealed that the relative abundance of *Lactobacillus* was significantly negatively correlated with the level of DCA and significantly positively correlated with the levels of HCA, HDCA, and MCA (Figure 4E).

At the KEGG level 2, compared with the HFD group, a total of nine metabolic pathways in the HDCA intervention group were significantly upregulated, including signal transduction, metabolism of cofactors and vitamins, amino acid metabolism, biosynthesis of other secondary metabolites, cellular processes and signaling, cellular motility, membrane transport, transcription, and the immune system. A total of 16 metabolic pathways were significantly downregulated, including glycan biosynthesis and metabolism, genetic information processing, cardiovascular diseases, translation, nucleotide metabolism, circulatory system, lipid metabolism, infectious diseases, endocrine system, cell growth and death, replication and repair, immune system diseases, metabolic diseases, signaling molecules and interactions, poorly characterized, and folding, sorting, and degradation (Figure 4F).

To further verify the microbiota of intestinal microorganisms in mice, we conducted qRT-PCR on mouse feces. The results showed that the expression levels of *Lactobacillus_johnsonii* and *L. reuteri* in the feces of mice in the HDCA group were significantly increased, which was consistent with our 16S results (Figure 4G).

## 3. Discussion

### 3.1. HDCA Regulates Bile Acid Metabolism via Deoxycholic Acid in CRC Mice

A high-fat diet is a major cause of obesity [1,15]. Excessive fat tissue accumulation can lead to fat tissue dysfunction, including lipid storage abnormalities, insulin resistance, ectopic lipid deposition, local hypoxia, and elevated ROS levels. These local changes are transmitted to multiple organs through altered secretomes, leading to adverse interactions between organs and promoting the onset and development of obesity-related diseases, including tumors [16].

Our previous research has already discovered the inhibitory effect of HDCA on obesity [11]. To investigate the effect of HDCA on the growth of colon cancer promoted by a high-fat diet, independent of its effect on body weight, we selected mice with similar body weights from the HFD group and the HFD supplemented HDCA group to conduct metabolic and microbiota studies. In human colorectal cancer patients, elevated levels of deoxycholic acid (DCA), taurodeoxycholic acid, glycocholic acid, and lithocholic acid have been observed, while ursodeoxycholic acid (UDCA) levels are reduced [17,18,19]. Our study found that HDCA regulated bile acid metabolism disorders in mice, increasing the content of HDCA and decreasing the content of DCA, which is consistent with these previous studies. In this study, we found that HDCA inhibits the progression of high-fat-diet-related colon cancer by reducing DCA. High-fat diets can cause obesity, which effects bile acid homeostasis, increasing secondary bile acids in feces and circulation. Long-term consumption of a high-fat diet leads to elevated levels of deoxycholic acid. A long-term high-fat diet can lead to increased levels of DCA [20,21,22]. DCA is a natural secondary bile acid produced by intestinal bacteria through the metabolism of bile acids and has potential carcinogenic and pro-inflammatory effects. It can promote the progression of colorectal cancer by DNA damage and the disruption to intestinal mucosal barrier [23,24,25,26,27]. In populations with a high incidence of colorectal cancer, the concentration of DCA in feces and serum increases [28].

### 3.2. HDCA Modulates Bile Acid Synthesis Pathways

The synthesis pathways of bile acids in the liver include the classical pathway, which is primarily mediated by CYP7A1, CYP8B1, and CYP27A1, and the alternative pathway, which is primarily mediated by CYP27A1 and CYP7B1. The classical pathway produces primary bile acids cholic acid (CA) and chenodeoxycholic acid (CDCA), while the alternative pathway catalyzes the production of CDCA. In rodent livers most CDCA is converted to α- and β-mouse bile acids (α-MCA and β-MCA), but in humans CDCA is not converted [29]. HDCA intervention has been found to alleviate human non-alcoholic fatty liver disease (NAFLD) by activating the alternative pathway centered on CYP7B1, inhibiting the classical pathway centered on CYP7A1, altering the bile acid synthesis pathway in the liver [13].

### 3.3. Gut Microbiota–Bile Acid Interactions in Antitumor Effects of HDCA

In this study, HDCA altered the composition of the mice gut microbiota. The relative abundance of *Lactobacillus* increased and showed a significant negative correlation with DCA levels while showing a significant positive correlation with HCA, HDCA, and MDCA levels. The metabolism of bile acids is closely related to the gut microbiota, and the interaction between bile acids and the gut microbiota affects the composition of each other. The gut microbiota participates in bile acid metabolism and conversion through decoupling, 7α/β-dehydroxylation, oxidation/isomerization, esterification, desulfurization, and coupling reaction. An imbalance in the gut microbiota disrupts bile acid metabolism and conversion, altering the concentration and composition of bile acid metabolites [30,31]. The types and levels of bile acids can also change the composition of the gut microbiota. For example, lithocholic acid (LCA) and its derivatives can exert antibacterial effects and inhibit the growth of harmful bacteria [32]. Supplement with bile acids leads to an increase in Phylum Firmicutes and a decrease in Bacteroidetes in rats [33]. High-fat-diet-induced obesity alters the composition of the gut microbiota, which can regulate the synthesis of secondary bile acids, such as *Bifidobacteria*, *Lactobacilli*, *Clostridia*, and *Enterococci*, which can convert unabsorbed bile acids into deoxycholic acid, lithocholic acid, and ursodeoxycholic acid [34]. The complex interaction between bile acids and the gut microbiota may play a role in the progression of colorectal cancer promoted by a high-fat diet. *Lactobacillus* can repair the intestinal epithelial barrier damaged by pathogenic bacteria and inhibit the proliferation of potential pathogens [35].

*Lactobacillus* regulate bile acid metabolism by secreting bile salt hydrolases (BSHs) that hydrolyze conjugated bile acids. In addition, *Lactobacillus* can activate Farnesoid X receptor (FXR), downregulate bile acid synthase, and regulate bile acid metabolism [36]. High levels of *Lactobacillus* in the intestine can reduce deoxycholic acid levels [37].

The interaction between gut microbiota and metabolites is very complex. This study speculates that HDCA supplementation may ameliorate HFD-induced obesity phenotypes by increasing the relative abundance of beneficial bacteria, such as *Lactobacillus*, thereby reshaping gut microbial composition. The HDCA-modulated gut microbiota may contribute to systemic metabolic effects through circulation, including a reduction in deoxycholic acid levels. Furthermore, HDCA turns liver bile acid synthesis from the classical pathway into the alternative pathway, characterized by decreased CYP7A1 and increased CYP7B1 expression. This coordinated modulation of gut microbiota and bile acid metabolism may represent a potential mechanism by which HDCA exerts protective effects against colorectal tumor development.

This study still has several limitations. First, the experimental design included only male mice from a single strain, which may limit the generalizability of our findings across sexes and genetic backgrounds. In addition, this study did not consider an orthotopic CRC model, and thus the tumor microenvironment may not fully recapitulate the physiological context of colorectal cancer. Due to practical and ethical constraints, the HDCA experiment included only three mice per group, which may limit the statistical robustness of the findings. Second, the study used HFD to induce obesity, but diet-induced metabolic alterations are inherently complex. The dietary effects may introduce additional confounding influences that are difficult to fully disentangle from the mechanisms under the investigation.

Although HDCA intervention resulted in reduced fecal α-diversity and influenced β-diversity, the study did not further explore the underlying drivers of these microbial alterations. Also in this study we observed a negative correlation between DCA levels and *Lactobacillus* abundance; however, the mechanistic basis by which DCA may suppress *Lactobacillus* was not examined. Similarly, we did not assess inflammatory pathways or immune profiling, both of which are likely to participate in the interaction between bile acids, microbiota, and CRC progression.

Future work should therefore aim to evaluate inflammation-related pathways and intestinal barrier-associated proteins. Incorporating orthotopic models, additional mice strains, and both sexes will further strengthen the findings and help refine the mechanism of HDCA’s protective effects against diet-induced CRC.

## 4. Materials and Methods

### 4.1. Animal Study

Animal experiments were approved by the ethics committee of Zhejiang University (ZJU 20240781, 4 October 2024) and performed following the guidelines of the center of Laboratory Animals at Zhejiang University (Hangzhou, China). Male C57BL/6J mice were purchased from Shanghai Slack Laboratory Animal Co., Ltd. (Shanghai, China). Male C57BL/6J mice (aged 5 weeks) were purchased from Charles River Laboratories (Jiaxing, China). All mice were housed in specific pathogen-free conditions.

#### 4.1.1. HDCA Intervention Experiment

For the HDCA supplementation experiment, 30 [11] male C57BL/6J mice (5 weeks) were randomly divided into three groups: the normal diet group (ND, 10% calories from fat), the high-fat diet group (HFD, 60% calories from fat), and the high-fat diet with an additional 0.5% hyodeoxycholic acid group (HFD+HDCA, 60% calories from fat). Weight and food intake were recorded once a week during the experiment.

After 21-week feeding, there was a significant difference in body weight between the ND group and the HFD group. The obesity model was successfully established. All mice feces were collected. MC38 tumor cells (1 × 10^6^ cells per mouse) were subcutaneously inoculated under the armpits of mice in the ND group (*n* = 4) and the mice with similar body weight in the HFD group (*n* = 3) and the HDCA group (*n* = 3). The body weight and tumor size were recorded weekly. Three weeks after inoculation, the feces of the mice were collected. Mice were fasted overnight and then sacrificed.

The harvested blood via cardiac puncture was centrifuged at 3500 rpm for 15 min for serum isolation. Liver, adipose tissues, intestinal tissues, and contents were collected immediately and kept in liquid nitrogen before storage at −80 °C.

#### 4.1.2. DCA Intervention Experiment

For the HDCA and DCA supplementation experiment, 30 5-week male C57BL/6J mice were adaptively fed for three weeks and then randomly divided into three groups: the high-fat diet group (HFD, 60% calories for fat, *n* = 10), an additional 0.5% hyodeoxycholic acid group (HFD+HDCA, 60% fat calories for fat, *n* = 10), a high-fat diet with an additional 0.5% hyodeoxycholic acid group, and a DCA water drinking group (HFD+HDCA+DCA, 60% fat calories for fat, 0.5%DCA aqueous solution, *n* = 10). Weight and food intake were recorded once a week during the experiment.

After 14-week feeding, there was a significant difference in body weight between the ND group and the HFD group. The obesity model was successfully established. MC38 tumor cells (1 × 10^6^ cells per mouse) were subcutaneously inoculated under the armpits of mice with similar body weight in the HFD group (*n* = 7), the HFD+HDCA group (*n* = 6), and the HFD+HDCA+DCA group (*n* = 6). Body weight and tumor size were recorded weekly.

Three weeks after inoculation, 12 mice (3 mice in each group) were randomly selected to be fasted overnight for intestinal barrier permeability experiment. The prepared fluorescein isothiocyanate dextran (FITC-dextran) solution was administered to the mice by gavage at a dose of 0.6 mg/g. After 4 h, blood was collected from the hearts of the anesthetized mice and centrifuged (3500 rpm, 15 min). The concentration of FITC-dextran in serum was detected by enzyme-linked immunosorbent assay with a fluorescence detection module. All mice were sacrificed. The harvested blood via cardiac puncture was centrifuged at 3500 rpm for 15 min for serum isolation. Liver, adipose tissues, intestinal tissues, and contents were collected immediately and kept in liquid nitrogen before storage at −80 °C.

### 4.2. BAs Analysis

Bile acids are the main components of bile and the final products of cholesterol metabolism through liver tissue. In this study, targeted metabolomics was conducted on mice feces and detected 45 kinds of bile acids. This study also conducted untargeted metabolomics on mice serum and targeted metabolomics including 33 kinds of bile acids. The sample extraction method and experimental procedure are shown in the Supplementary Materials.

### 4.3. RNA Isolation and Quantitative Reverse Transcription PCR

#### 4.3.1. Liver RNA Isolation and qRT-PCR

Total Liver RNA was extracted using a total RNA extraction kit (Tiangen Biotech, Beijing, China), according to the manufacturer’s instructions. qRT-PCR was performed with a LightCycler 480 instrument (Roche, Basel, Switzerland) with the following primers: mouse CYP7A1, F, 5′-GCTGTGGTAGTGAGCTGTTG-3′, R, 5′-GTTGTCCAAAGGAGGTTCACC-3′; mouse CYP27A1, F, 5′-TCGCACCAATGTGAATCTGGCTAG-3′, R, 5′-CTTCCACTGCTCCATGCTGTCTC-3′; mouse CYP7B1, F, 5′-GGAGCCACGACCCTAGATG-3′, R, 5′-GCCATGCCAAGATAAGGAAGC-3′; mouse GAPDH, F, 5′-TGAACGGGAAGCTCACTGG-3′, R, 5′-TCCACCACCCTGTTGCTGTA-3′.

Targeted gene levels were normalized to housekeeping gene levels (GAPDH) and the results were analyzed using the ΔΔCT analysis method [38].

#### 4.3.2. Fecal RNA Isolation and qRT-PCR

Total fecal RNA was isolated using Fecal Genomic DNA Extraction Kit (Tiangen Biotech, Beijing, China), according to the manufacturer’s instructions. qRT-PCR was performed with a LightCycler 480 instrument (Roche, Basel, Switzerland) with the following primers: Lactobacillus_johnsonii Sense, 5′-CCTTGTCATTAGTTGCCATC-3′, Lactobacillus_johnsonii AntiSense, 5′-GGTTCGCTTCTCGTTGTA-3′; L.reuteri Sense, 5′-GGTTGCTTAGGTCTGATGT-3′, L.reuteri AntiSense, 5′-GGAGTGCTTAATGCGTTAG-3′; mouse β-actin, F, 5′-CGTTGACATCCGTAAAGAC-3′, R, 5′-AACAGTCCGCCTAGAAGCAC-3′. Targeted gene levels were normalized to housekeeping gene levels (β-actin), and the results were analyzed using the ΔΔCT analysis method [38].

### 4.4. 5R 16S rRNA Sequencing and Data Analysis

The stool samples of mice were collected under sterile conditions and immediately stored at −80 °C. Total genome DNA from samples was extracted using CTAB method [39].

16S rRNA gene amplification and sequencing was performed by amplifying five regions on the 16S rRNA gene in multiplex. The libraries were sequenced on Illumina NovaSeq 6000 system. Reads were demultiplexed per sample, filtered, and aligned to each of the five amplified regions based on the primers’ sequences. The Short MUlitiple Regions Framework (SMURF) method was applied to combine read counts from the five regions into a coherent profiling results solving a maximum likelihood problem [40]. The GreenGenes database (May 2013 version, with some improvements) was used as a reference.

### 4.5. Statistical Analysis

Results are presented as mean ± SEM. Statistical significance was analyzed using the unpaired Student’s *t*-test and One-way ANOVA. The Mann–Whitney U test was used for intestinal permeability test analysis for data with a non-normal distribution. Statistical analyses were calculated using GraphPad prism 9.0 (GraphPad software, La Jolla, CA, USA) and R 4.4.1 (RStudio, Boston, MA, USA). Correlations between BAs and microbiome abundances were performed using Spearman’s correlation analysis with the post hoc correction using the FDR method. Principal co-ordinates analysis (PCoA) and heatmaps depicting spearman correlation were generated using R 4.1.1 (RStudio, Boston, MA, USA). Differences between experimental groups were considered significant at *p* < 0.05.

## 5. Conclusions

This study hypothesizes that HDCA supplementation may ameliorate HFD-induced obesity phenotypes by altering the composition of the gut microbiota. The HDCA-modulated microbial community may exert systemic metabolic effects through the circulation, including decreasing deoxycholic acid levels. HDCA tends to shift liver bile acid synthesis from the classical pathway toward the alternative pathway by decreased CYP7A1 expression and increased CYP7B1 expression. This coordinated modulation of the gut microbiota and bile acid metabolism may represent a potential mechanism through which HDCA confers protective effects against CRC.

## Data Availability

The data that support the findings of this study are openly available in Sequence Read Archive at https://www.ncbi.nlm.nih.gov/sra/PRJNA1334311 (accessed on 30 November 2025), reference number PRJNA1334311.

## Supplementary Materials

The following supporting information can be downloaded at https://www.mdpi.com/article/10.3390/nu17243939/s1, File S1: Methods of bile acids analysis.

## References

[1] Tong Y., Gao H., Qi Q., Liu X., Li J., Gao J., Li P., Wang Y., Du L., Wang C. (2021). High Fat Diet, Gut Microbiome and Gastrointestinal Cancer. Theranostics.

[2] Huang M., Narita S., Koizumi A., Nara T., Numakura K., Satoh S., Nanjo H., Habuchi T. (2021). Macrophage Inhibitory Cytokine-1 Induced by a High-Fat Diet Promotes Prostate Cancer Progression by Stimulating Tumor-Promoting Cytokine Production from Tumor Stromal Cells. Cancer Commun..

[3] Wang Y., Yan F., Chen Q., Liu F., Xu B., Liu Y., Huo G., Xu J., Li B., Wang S. (2024). High-Fat Diet Promotes Type 2 Diabetes Mellitus by Disrupting Gut Microbial Rhythms and Short-Chain Fatty Acid Synthesis. Food Funct..

[4] Chen J., Xiao Y., Li D., Zhang S., Wu Y., Zhang Q., Bai W. (2023). New Insights into the Mechanisms of High-Fat Diet Mediated Gut Microbiota in Chronic Diseases. Imeta.

[5] Gonzalez-Gutierrez L., Motiño O., Barriuso D., de la Puente-Aldea J., Alvarez-Frutos L., Kroemer G., Palacios-Ramirez R., Senovilla L. (2024). Obesity-Associated Colorectal Cancer. Int. J. Mol. Sci..

[6] Morgan E., Arnold M., Gini A., Lorenzoni V., Cabasag C.J., Laversanne M., Vignat J., Ferlay J., Murphy N., Bray F. (2023). Global Burden of Colorectal Cancer in 2020 and 2040: Incidence and Mortality Estimates from GLOBOCAN. Gut.

[7] Papier K., Bradbury K.E., Balkwill A., Barnes I., Smith-Byrne K., Gunter M.J., Berndt S.I., Le Marchand L., Wu A.H., Peters U. (2025). Diet-Wide Analyses for Risk of Colorectal Cancer: Prospective Study of 12,251 Incident Cases among 542,778 Women in the UK. Nat. Commun..

[8] Lauby-Secretan B., Scoccianti C., Loomis D., Grosse Y., Bianchini F., Straif K. (2016). Body Fatness and Cancer—Viewpoint of the IARC Working Group. N. Engl. J. Med..

[9] Mandic M., Safizadeh F., Niedermaier T., Hoffmeister M., Brenner H. (2023). Association of Overweight, Obesity, and Recent Weight Loss With Colorectal Cancer Risk. JAMA Netw. Open.

[10] Rohr M.W., Narasimhulu C.A., Rudeski-Rohr T.A., Parthasarathy S. (2020). Negative Effects of a High-Fat Diet on Intestinal Permeability: A Review. Adv. Nutr..

[11] Wang C., Lin J., Duan M., He J., Halizere S., Chen N., Chen X., Jiao Y., He W., Dyar K.A. (2025). Multi-Omics Reveals Different Signatures of Obesity-Prone and Obesity-Resistant Mice. iMetaOmics.

[12] Sehayek E., Ono J.G., Duncan E.M., Batta A.K., Salen G., Shefer S., Neguyen L.B., Yang K., Lipkin M., Breslow J.L. (2001). Hyodeoxycholic Acid Efficiently Suppresses Atherosclerosis Formation and Plasma Cholesterol Levels in Mice. J. Lipid Res..

[13] Kuang J., Wang J., Li Y., Li M., Zhao M., Ge K., Zheng D., Cheung K.C.P., Liao B., Wang S. (2023). Hyodeoxycholic Acid Alleviates Non-Alcoholic Fatty Liver Disease through Modulating the Gut-Liver Axis. Cell Metab..

[14] Pang Q., Huang S., Li X., Cao J. (2025). Hyodeoxycholic Acid Inhibits Colorectal Cancer Proliferation through the FXR/EREG/EGFR Axis. Front. Cell Dev. Biol..

[15] Wen X., Feng X., Xin F., An R., Huang H., Mao L., Liu P., Zhang J., Huang H., Liu X. (2024). *B. vulgatus* Ameliorates High-Fat Diet-Induced Obesity through Modulating Intestinal Serotonin Synthesis and Lipid Absorption in Mice. Gut Microbes.

[16] Sanhueza S., Simón L., Cifuentes M., Quest A.F.G. (2023). The Adipocyte-Macrophage Relationship in Cancer: A Potential Target for Antioxidant Therapy. Antioxidants.

[17] Cai J., Sun L., Gonzalez F.J. (2022). Gut Microbiota-Derived Bile Acids in Intestinal Immunity, Inflammation, and Tumorigenesis. Cell Host Microbe.

[18] Kühn T., Stepien M., López-Nogueroles M., Damms-Machado A., Sookthai D., Johnson T., Roca M., Hüsing A., Maldonado S.G., Cross A.J. (2020). Prediagnostic Plasma Bile Acid Levels and Colon Cancer Risk: A Prospective Study. J. Natl. Cancer Inst..

[19] Bernstein C., Holubec H., Bhattacharyya A.K., Nguyen H., Payne C.M., Zaitlin B., Bernstein H. (2011). Carcinogenicity of Deoxycholate, a Secondary Bile Acid. Arch. Toxicol..

[20] Li R., Andreu-Sánchez S., Kuipers F., Fu J. (2021). Gut Microbiome and Bile Acids in Obesity-Related Diseases. Best Pract. Res. Clin. Endocrinol. Metab..

[21] Alonso N., Almer G., Semeraro M.D., Rodriguez-Blanco G., Fauler G., Anders I., Ritter G., Vom Scheidt A., Hammer N., Gruber H.-J. (2024). Impact of High-Fat Diet and Exercise on Bone and Bile Acid Metabolism in Rats. Nutrients.

[22] Fang Y., Yan C., Zhao Q., Xu J., Liu Z., Gao J., Zhu H., Dai Z., Wang D., Tang D. (2021). The Roles of Microbial Products in the Development of Colorectal Cancer: A Review. Bioengineered.

[23] Liu L., Dong W., Wang S., Zhang Y., Liu T., Xie R., Wang B., Cao H. (2018). Deoxycholic Acid Disrupts the Intestinal Mucosal Barrier and Promotes Intestinal Tumorigenesis. Food Funct..

[24] Yoshimoto S., Loo T.M., Atarashi K., Kanda H., Sato S., Oyadomari S., Iwakura Y., Oshima K., Morita H., Hattori M. (2013). Obesity-Induced Gut Microbial Metabolite Promotes Liver Cancer through Senescence Secretome. Nature.

[25] Cong J., Liu P., Han Z., Ying W., Li C., Yang Y., Wang S., Yang J., Cao F., Shen J. (2024). Bile Acids Modified by the Intestinal Microbiota Promote Colorectal Cancer Growth by Suppressing CD8+ T Cell Effector Functions. Immunity.

[26] Zhao S., Gong Z., Du X., Tian C., Wang L., Zhou J., Xu C., Chen Y., Cai W., Wu J. (2018). Deoxycholic Acid-Mediated Sphingosine-1-Phosphate Receptor 2 Signaling Exacerbates DSS-Induced Colitis through Promoting Cathepsin B Release. J. Immunol. Res..

[27] Bernstein H., Bernstein C., Payne C.M., Dvorak K. (2009). Bile Acids as Endogenous Etiologic Agents in Gastrointestinal Cancer. World J. Gastroenterol..

[28] Ocvirk S., O’Keefe S.J.D. (2021). Dietary Fat, Bile Acid Metabolism and Colorectal Cancer. Semin. Cancer Biol..

[29] Jia W., Xie G., Jia W. (2018). Bile Acid-Microbiota Crosstalk in Gastrointestinal Inflammation and Carcinogenesis. Nat. Rev. Gastroenterol. Hepatol..

[30] Pan Y., Zhang H., Li M., He T., Guo S., Zhu L., Tan J., Wang B. (2024). Novel Approaches in IBD Therapy: Targeting the Gut Microbiota-Bile Acid Axis. Gut Microbes.

[31] Wei M., Huang F., Zhao L., Zhang Y., Yang W., Wang S., Li M., Han X., Ge K., Qu C. (2020). A Dysregulated Bile Acid-Gut Microbiota Axis Contributes to Obesity Susceptibility. eBioMedicine.

[32] do Nascimento P.G.G., Lemos T.L.G., Almeida M.C.S., de Souza J.M.O., Bizerra A.M.C., Santiago G.M.P., da Costa J.G.M., Coutinho H.D.M. (2015). Lithocholic Acid and Derivatives: Antibacterial Activity. Steroids.

[33] Islam K.B.M.S., Fukiya S., Hagio M., Fujii N., Ishizuka S., Ooka T., Ogura Y., Hayashi T., Yokota A. (2011). Bile Acid Is a Host Factor That Regulates the Composition of the Cecal Microbiota in Rats. Gastroenterology.

[34] Guzior D.V., Quinn R.A. (2021). Review: Microbial Transformations of Human Bile Acids. Microbiome.

[35] Zhao X., Jiang L., Fang X., Guo Z., Wang X., Shi B., Meng Q. (2022). Host-Microbiota Interaction-Mediated Resistance to Inflammatory Bowel Disease in Pigs. Microbiome.

[36] Wu L., Zhou J., Zhou A., Lei Y., Tang L., Hu S., Wang S., Xiao X., Chen Q., Tu D. (2024). Lactobacillus Acidophilus Ameliorates Cholestatic Liver Injury through Inhibiting Bile Acid Synthesis and Promoting Bile Acid Excretion. Gut Microbes.

[37] Sánchez B. (2018). Bile Acid-Microbiota Crosstalk in Gastrointestinal Inflammation and Carcinogenesis: A Role for Bifidobacteria and Lactobacilli?. Nat. Rev. Gastroenterol. Hepatol..

[38] Livak K.J., Schmittgen T.D. (2001). Analysis of Relative Gene Expression Data Using Real-Time Quantitative PCR and the 2(-Delta Delta C(T)) Method. Methods.

[39] Nejman D., Livyatan I., Fuks G., Gavert N., Zwang Y., Geller L.T., Rotter-Maskowitz A., Weiser R., Mallel G., Gigi E. (2020). The Human Tumor Microbiome Is Composed of Tumor Type-Specific Intracellular Bacteria. Science.

[40] Fuks G., Elgart M., Amir A., Zeisel A., Turnbaugh P.J., Soen Y., Shental N. (2018). Combining 16S rRNA Gene Variable Regions Enables High-Resolution Microbial Community Profiling. Microbiome.

