# Supplementary material for: Hyodeoxycholic Acid Suppresses High-Fat-Diet–Promoted MC38-Syngeneic Colorectal Tumor Growth via Bile Acid Remodeling and Microbiota Modulation
Source: Nutrients. 2025 Dec 17;17(24):3939. doi: 10.3390/nu17243939 (PMC12735938; doi:10.3390/nu17243939)
Supplement: Supplementary file 1 [file nutrients-17-03939-s001.zip › nutrients-3996942-supplementary.pdf]

Methods of bile acids analysis

1. Fecal Bile Acid Analysis

1.1 Sample preparation

Samples are accurately weighed 20~50.0 mg in EP tube under ice conditions. After adding two steel ball, the samples were mixed with 500 μL of 80% methanol water for protein precipitation. The mixture was grinded for 3 min and vortexed for 20 min before centrifugation(4℃，20000 rcf）for 15 min. The supernatant was collected for LC-MS/MS analysis.

1.2 LC-MS/MS Analysis

Thermo Scientific TSQ Altis liquid chromatography-tandem mass spectrometry system, coupled with the Vanquish liquid chromatography system.

The chromatographic separation was performed on a Agilent EclipsePlus C18 RRHD(3.0 * 150 mm，1.8 μm). The mobile phase consisted of 10 mmol/L ammonium acetate (A) and mixed liquor of methanol and acetonitrile (B), at a total flow rate of 0.45 mL/min. The column temperature was set at 40°C. The injection volume of all samples was 5 μL.

For MS detection, the system was operated in multiple reaction monitoring(MRM) mode and used an electrospray ionization(ESI) probe as an ion source in negative polarity.

1.3 Chemical experiment reagent consumables

Reagent	Manufacturers	Specification	Lot
Methanol	CNW	4L/ bottle	CAEQ-4-000306-4000
Acetonitrile	CNW	4L/ bottle	CAEQ-4-000308-4000
Ammonium acetate	CNW	50g/bottle	CAEQ-4-000314-0050

2. Serum Targeted Metabolomics-Materials and Methods

2.1 Reagents and instruments

Equipments: An ultra-high performance liquid chromatography coupled to tandem mass spectrometry (UHPLC-MS/MS) system (ExionLC™ AD UHPLC-QTRAP® 6500+, AB SCIEX Corp., Boston, MA, USA).

Materials and reagents: All of the 33 bile acid standards and 6 stable isotope-labeled standards were obtained from ZZ Standards Co., LTD. (Shanghai, China). Ammonium acetate was of analytical grade and obtained from Sigma-Aldrich (St. Louis, MO, USA). Methanol (Optima LC-MS), acetonitrile (Optima LC-MS), and formic acid (Optima LC-MS) were purchased from Thermo-Fisher Scientific (FairLawn, NJ, USA).

2.2 Standard solution preparation

The stock solution of individual bile acid was mixed and prepared in bile acid-free matrix to obtain a series of bile acid calibrators at a concentration of 25000、15000、5000、2500、500、250、50、25、15、5、2.5 or 1.5 ng/mL. Certain concentrations of GCA-d4, UDCA-d4, CA-d4, GCDCA-d4, LCA-d4 and CDCA-d4 were compounded and mixed as Internal Standard (IS). The stock solution of all of these and working solution were stored in refrigerator of -20°C.

2.3 Metabolites extraction

The samples were added to water by well vortexing as the diluted sample. Then 100 μL of it was taken and homogenized with 300 μL of acetonitrile/methanol (8:2) which contained mixed internal standards by well vortexing. Next, put it on ice for 30min. After that, centrifuged at 12,000 rpm for 10 min. Finally, the supernatant was injected into the LC-MS/MS system for analysis.

2.4 LC-MS method

An ultra-high performance liquid chromatography coupled to tandem mass spectrometry (UHPLC-MS/MS) system (ExionLC™ AD UHPLC-QTRAP 6500+, AB SCIEX Corp., Boston, MA, USA) was used to quantitate bile acids in Novogene Co., Ltd. (Beijing, China). Separation was performed on a Waters ACQUITY UPLC BEH C18 column (2.1×100mm, 1.7μm) which was maintained at 50°C. The mobile phase, consisting of 0.1% formic acid in water (solvent A) and acetonitrile (solvent B), was delivered at a flow rate of 0.30 mL/min. The solvent gradient was set as follows: initial 20% B, 0.5min; 20-35% B, 1min; 35-37% B, 2.5 min; 37-38% B, 4.1min; 38-39% B, 6min; 39-40% B, 6.5min; 40-44% B, 8.5min; 44-45% B, 9min; 45-52% B, 9.5min; 52-65% B, 12.5min; 65-100% B, 13min; 100-20% B, 15.1min; 20% B, 17 min.

The mass spectrometer was operated in negative multiple reaction mode (MRM) mode. Parameters were as follows: IonSpray Voltage (-4500 V), Curtain Gas (35 psi), Ion Source Temp (550°C), Ion Source Gas of 1 and 2 (60 psi).

2.5 Standard curve and LOQ

LC-MS was used to detect the concentration series of standard solution. The ratio of concentration of standard to internal standard as abscissa, and the ratio of peak area of standard to internal standard as ordinate to investigate the linearity of standard solution. The correlation coefficient (r) > 0.99 of each metabolites were the necessary condition. The limit of quantification (LOQ) were determined by the method of signal-to-noise ratio (S/N), which is comparing the signal measured by the standard solution concentration with the blank matrix. Generally, when the S/N = 10:1, the corresponding concentration is the LOQ.

2.6 Matrix effects

Matrix refers to the components other than the target in the sample. Matrix often has significant interference on the analysis process of the target and affects the accuracy of the analysis results, these effects and interferences are called matrix effects. To evaluate the matrix effects of bile acids on the ionization of the analyze, its value can be calculated as follows: ME% = {(QC matrix - B blank) / QC MS water-1} × 100%. ME is matrix effect, QC matrix is blank matrix with standard sample, QC MS water is matrix free with standard sample, and B blank is blank matrix sample. The matrix effects results showed that the matrix effects was less in the range of - 20% to 20%, moderate in the range of - 20% to - 50% or 20% to 50%, and greater in the range of less than - 50% or more than 50%.

2.7 Precision

Precision reflects the degree of consistency between independent measurement results, including intra-day precision (repeatability) and inter-day precision (reproducibility). The precision for this method was determined at three different concentration levels on three days. The intra-day and inter-day precision require to RSD ≤ 15%.

2.8 Accuracy

Accuracy refers to the closeness of the measured results with the reference value. Usually expressed in terms of the recovery of the sample. The accuracy for this method can be calculated as follows: R% = (QC recovery sample - B blank) / S × 100%. R% is recovery rate, QC recovery sample is matrix with recovery point sample, B blank is blank matrix sample, S is theoretical concentration. The accuracy for this method was determined at three different concentration levels. The accuracy requirement 85% to 115% and RSD ≤ 15% of three different concentration levels.

2.9 Stability

Stability mainly evaluates the stability of the target in the biological matrix and the stability of the target placed after sample treatment. This method mainly evaluates the stability of the target substance in the

injector (4°C) for 24 hours after sample treatment. The acceptance standard was the RSD ≤ 15% of each target within 24 hours is.

3. Serum Untargeted Metabolomics-Materials and Methods

3.1 Metabolites Extraction

The liquid samples (100 µL) were placed in the EP tubes and resuspended with prechilled 80% methanol by well vortex. Then the samples were incubated on ice for 5 min and centrifuged at 15,000 g, 4°C for 20 min. Some of supernatant was diluted to final concentration containing 53% methanol by LC-MS grade water. The samples were system analysis.

3.2 UHPLC-MS/MS Analysis

UHPLC-MS/MS analyses were performed using a Vanquish UHPLC system (ThermoFisher, Germany) coupled with an Orbitrap Q ExactiveTM HF mass spectrometer or Orbitrap Q ExactiveTMHF-X mass spectrometer (Thermo Fisher, Germany) in Novogene Co., Ltd. (Beijing, China). Samples were injected onto a Hypersil Goldcolumn (100×2.1 mm, 1.9µm) using a 12-min linear gradient at a flow rate of 0.2 mL/min. The eluents for the positive and negative polarity modes were eluent A (0.1% FA in Water) and eluent B (Methanol). The solvent gradient was set as follows: 2% B, 1.5 min; 2-85% B, 3 min; 85-100% B, 10 min；100-2% B, 10.1 min；2% B, 12 min. Q ExactiveTM HF mass spectrometer was operated in positive/negative polarity mode with spray voltage of 3.5 kV, capillary temperature of 320°C, sheath gas flow rate of 35 psi and aux gas flow rate of 10 L/min, S-lens RF level of 60, Aux gas heater temperature of 350°C.

3.3 Data processing and metabolite identification

The raw data files generated by UHPLC-MS/MS were processed using the Compound Discoverer 3.3 (CD3.3, ThermoFisher) to perform peak alignment, peak picking, and quantitation for each metabolite. The main parameters were set as follows: peak area was corrected with the first QC, actual mass tolerance, 5ppm; signal intensity tolerance, 30%; and minimum intensity, et al. After that, peak intensities were normalized to the total spectral intensity. The normalized data was used to predict the molecular formula based on additive ions, molecular ion peaks and fragment ions. And then peaks were matched with the mzCloud (https://www.mzcloud.org/), mzVault and MassList database to obtain the accurate qualitative and relative quantitative results. Statistical analyses were performed using the statistical software R (R version R-3.4.3), Python (Python 2.7.6 version) and CentOS (CentOS release 6.6), When data were not normally distributed, standardize according to the formula: sample raw quantitation value / (The sum of sample metabolite quantitation value / The sum of QC1 sample metabolite quantitation value) to obtain relative peak areas; And compounds whose CVs of relative peak areas in QC samples were greater than 30% were removed, and finally the metabolites' identification and relative quantification results were obtained.

3.4 Data Analysis

These metabolites were annotated using the KEGG database (https://www.genome.jp/kegg/pathway.html)，HMDB database (https://hmdb.ca/ metabolites) and LIPIDMaps database (http://www.lipidmaps.org/). Principal components analysis (PCA) and Partial least squares discriminant analysis (PLS-DA) were performed at metaX(a flexible and comprehensive software for processing metabolomics data).We applied univariate analysis (t-test) to calculate the statistical significance (P-value).The metabolites with VIP > 1 and P-value< 0.05 and fold change≥2 or FC≤0.5 were considered to be differential metabolites. Volcano plots were used to filter metabolites of interest which based on log_2(FoldChange) and -log_{10}(p-value) of metabolites by ggplot2 in R language.

For clustering heat maps, the data were normalized using z-scores of the intensity areas of differential metabolites and were ploted by Pheatmap package in R language. The correlation between differential metabolites were analyzed by cor () in R language (method=pearson). Statistically significant of correlation between differential metabolites were calculated by cor.mtest() in R language. P-value < 0.05 was considered as statistically significant and correlation plots were ploted by corrplot package in R language. The functions of these metabolites and metabolic pathways were studied using the KEGG database. The metabolic pathways enrichment of differential metabolites was performed, when ratio were satisfied by $x/n > y/N$, metabolic pathway were considered asenrichment, when P-value of metabolic pathway < 0.05, metabolic pathway were considered as statistically significant enrichment.
